# Death from Chloroform

**Published:** 1849-03

**Authors:** 


*Death from Chloroform.*—A coroner's inquest was held in New York, on the 20th of January, on the body of a seaman, thirty-one years of age, who came to his death the previous day by the effects of chloroform, while undergoing an operation in the New York Hospital.

Dr. Buck, the attending surgeon, in his evidence, stated that, "on or about the 26th of December, I advised that chloroform should be administered to the deceased for the purpose of examining the condition of the rectum, the parts being in such a state of excessive irritability as scarcely to admit of a separation of the nates. The patient recovered from the effects of the chloroform, and remained in all respects in the same condition as before its use. On the 19th of January, the deceased being in a sound condition, except the local ailments spoken of, and he having never complained of either his head or chest, and not having suffered from the first administration of chloroform, I directed it to be administered to him for the purpose of performing an operation upon the rectum, and the operation of circumcision to remove a phymosis caused by the chancres: the patient soon became excited by the chloroform, as is usually the case, but not beyond a degree which I have often observed. At this moment my attention was arrested by my assistant calling for a wet cloth. On examining the patient I found his face and neck of a livid leaden hue or color, the eyes tumid, the pulse imperceptible at the wrist, and the whole body relaxed; after two or three gasps he ceased to breathe; every means were promptly used to restore the deceased, but without effect. The chloroform was obtained at Lent's, 91 John Street, and not exceeding three drachms was administered from a napkin; a portion of chloroform from the same vial had been administered the day before, to a patient without any unfavorable effects: about ten minutes elapsed from the commencement of its administration before death took place. On making a post-mortem examination twenty-four hours after death, I found the face less livid than before death; on examining the head, the brain and its membranes presented no other appearance than are seen in persons dying when in full health; the lungs were a good deal congested; and discharged, when cut, a large quantity of bloody serum; the heart was large, its ventricles and auricles were empty; its condition flabby; the substance of the left ventricle rather softer than natural; about half an ounce of a watery fluid was found in the pericardium; the viscera of the abdomen were healthy.—*Western Journal of Medicine and Surgery.*

